# Supplementary material for: Step-by-step causal analysis of EHRs to ground decision-making
Source: PLOS Digit Health. 2025 Feb 3;4(2):e0000721. doi: 10.1371/journal.pdig.0000721 (PMC11790099; doi:10.1371/journal.pdig.0000721)
Supplement: S1 Fig — (PDF) [file pdig.0000721.s001.pdf]

Supporting information

S1 Fig Motivating example: Failure of predictive models to predict mortality from pretreatment variables. To illustrate how machine learning frameworks can fail to inform decision making, we present a motivating example from MIMIC-IV. Using the same population and covariates as in the main analysis (described in S1 Table), we train a predictive model for 28-day mortality. We split the data into a training set (80%) and a test set (20%). The training set uses the last measurements from the first 24 hours, whereas the validation set only uses the last measurements before the administration of crystalloids. We split the train set into a train and a validation set. We fit a HistGradientBoosting classifier (https://scikit-learn.org/stable/modules/ensemble.html#histogram-based-gradient-boosting) on the train set and evaluate the performance on the validation set and on the test set. We see good area under the Precision-recall curve (PR AUC) on the validation set, but a deterioration of 10 points on the test set (Fig 1a). The same is seen in Fig 1b when measuring performances with Area Under the Curve of the Receiving Operator Characteristic (ROC AUC). On the contrary, a model trained on pre-treatment features yields competitive performances. This failure illustrates well the shortcuts on which predictive models could rely to make predictions. A clinically useful predictive model should support decision-making –in this case, addition of albumin to crystalloids– rather than maximizing predictive performance. In this example, causal thinking would have helped to identify the bias introduced by post-treatment features. In fact, these features should not be included in a causal analysis since they are post-treatment colliders.

This kind of error might sound naive to a clinical expert but relying on shortcuts –some of them being post-treatment variables– is a common error. Here, we detail some real use cases where machine learning fail in providing useful predictions for decision-making. [1] use deep learning to predict hip fracture using confounding patient and healthcare variables. An example of such covariates shown by the authors is the triage of patients before imaging that results in the model trying to predict the image acquisition machine and rely on it to predict hip fracture. [2] describe the use of algorithm in US extra-care programs. By equating care needs with previous care costs (in a pure predictive fashion), the algorithm falsely conclude that Black patients are healthier than equally white patients, since they do less money is spent on them for a given level of need. Beyond Machine Learning, we also spotted the inclusion of post-treatment variables in the development of the recent SCORE2 cardio-vascular risk score [3]: *Our risk models might have underestimated CVD risk be- cause data used to estimate multipliers were likely to include some people already on CVD prevention therapies (e.g. statins or anti- hypertensive medication.* This score might be used to inform on the initiation of statins for primary prevention. But, relying on post-treatment, it might under-discover patients who would benefit from statins at screening time.

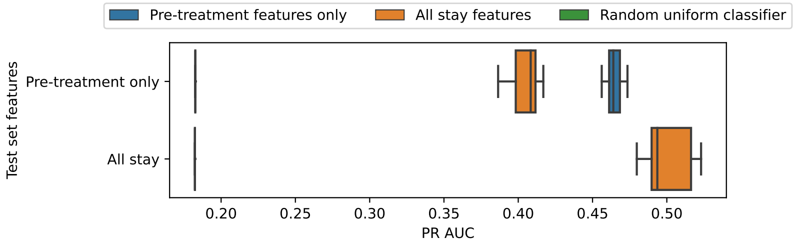

(a) *Area under the Precision-Recall curve (PR_AUC)*

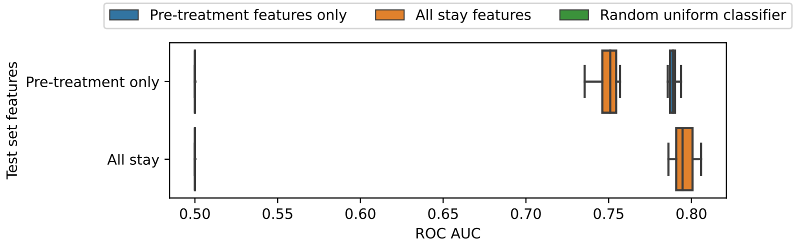

(b) *Area under the Receiving Operator Characteristic (ROC_AUC)*

Fig 1. ***Failure to predict 28-day mortality from a model fitted on pre-treatment variables.***
The model is trained on the last features from the whole stay and tested on two validation sets: one with all stay features and one with last features before crystalloids administration (Pre-treatment only). The all-stay model performance markedly decreases in the pre-treatment only dataset.

References

1. Badgeley MA, Zech JR, Oakden-Rayner L, Glicksberg BS, Liu M, Gale W, et al. Deep learning predicts hip fracture using confounding patient and healthcare variables. NPJ digital medicine. 2019;2(1):31.

2. Obermeyer Z, Powers B, Vogeli C, Mullainathan S. Dissecting racial bias in an algorithm used to manage the health of populations. Science. 2019;366(6464):447–453.

3. working group S. SCORE2 risk prediction algorithms: new models to estimate 10-year risk of cardiovascular disease in Europe. European heart journal. 2021;42(25):2439–2454.
