# Supplementary material for: Step-by-step causal analysis of EHRs to ground decision-making
Source: PLOS Digit Health. 2025 Feb 3;4(2):e0000721. doi: 10.1371/journal.pdig.0000721 (PMC11790099; doi:10.1371/journal.pdig.0000721)
Supplement: S2 Fig — (PDF) [file pdig.0000721.s002.pdf]

Supporting information

S2 Fig Immortal time bias illustration.

Fig 1 illustrates the immortal time bias. This time bias is a major pitfall in the retrospective evaluation of screening programs [1].

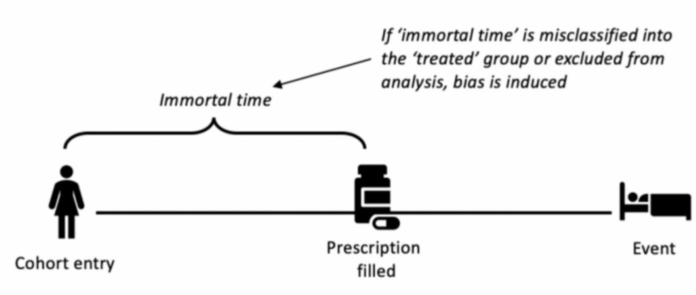

Fig 1. Immortal time bias illustration.
Poor experimental design can introduce Immortal time bias, which leads to a treated group with falsely longer longevity [2].

References

1. Bretthauer M, Kalager M. Principles, effectiveness and caveats in screening for cancer. Journal of British Surgery. 2013;100(1):55–65.

2. Lee H, Nunan D. Immortal time bias, Catalogue of Bias Collaboration.; 2020. Available from: https://catalogofbias.org/biases/immortaltimebias/.
