# Supplementary material for: Step-by-step causal analysis of EHRs to ground decision-making
Source: PLOS Digit Health. 2025 Feb 3;4(2):e0000721. doi: 10.1371/journal.pdig.0000721 (PMC11790099; doi:10.1371/journal.pdig.0000721)
Supplement: S3 Fig — (PDF) [file pdig.0000721.s003.pdf]

Supporting information

S3 Fig Graphical timeline. Drawing a graphical timeline as the one in Fig 1 during the study design helps to detect and prevent time-related biases.

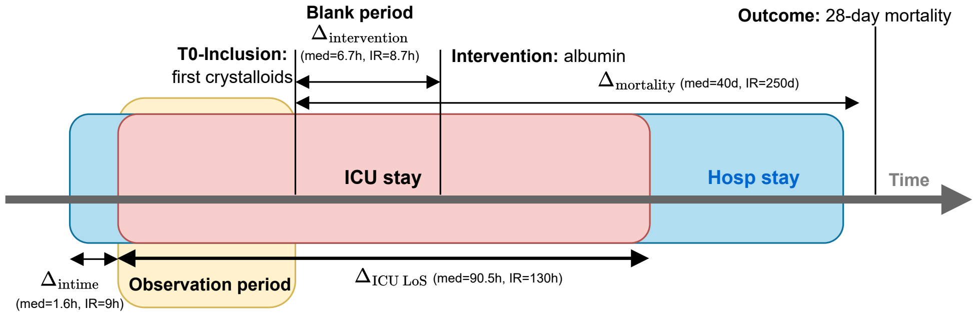

Fig 1. Graphical timeline.
Defining the inclusion event, the starting time T0 for follow-up, the intervention's assignment time and the observation window for confounders is crucial to avoid time and selection biases. In our study, the gap between the intervention and the inclusion is small compared to the occurrence of the outcome to limit immortal time bias: 6.7 hours vs 40 days for mortality.
