# Supplementary material for: Step-by-step causal analysis of EHRs to ground decision-making
Source: PLOS Digit Health. 2025 Feb 3;4(2):e0000721. doi: 10.1371/journal.pdig.0000721 (PMC11790099; doi:10.1371/journal.pdig.0000721)
Supplement: S4 Fig — (PDF) [file pdig.0000721.s004.pdf]

Supporting information

S4 Fig. Types of causal variables.

Fig 1 illustrates the different types of causal variables.

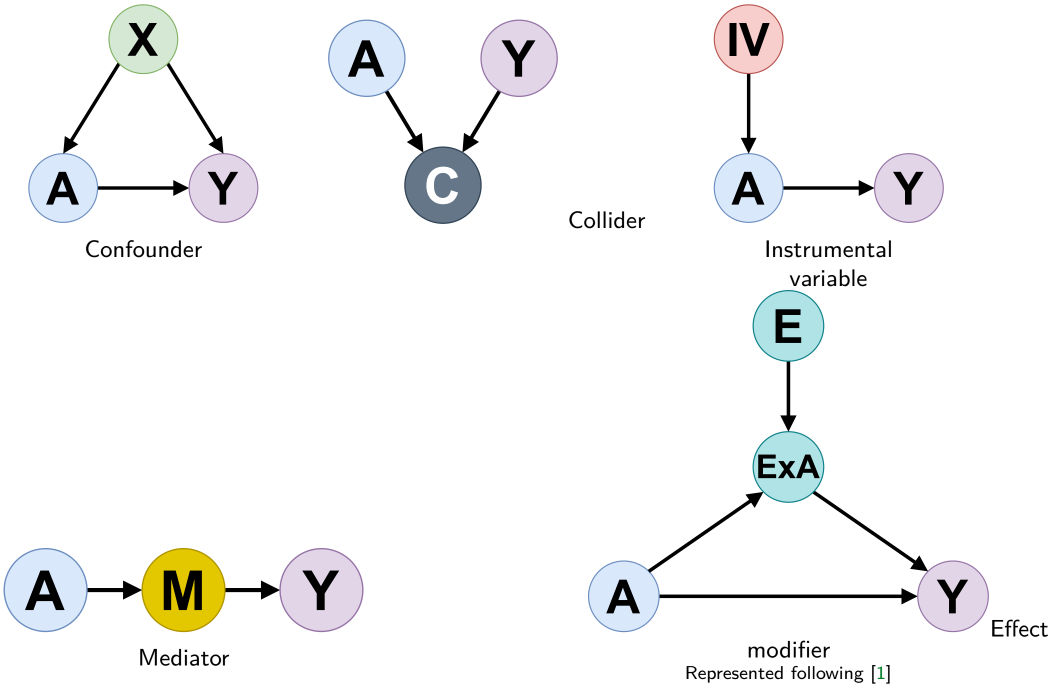

Fig 1. ***The five categories of causal variables needed for our framework.*** *A: Treatment, X: Confounder, IV: Instrumental variable, M: mediator, Y: Outcome, C: Collider, E: Effect modifier.*

References

1. Attia J, Holliday E, Oldmeadow C. A proposal for capturing interaction and effect modification using DAGs; 2022.
