# Supplementary material for: Step-by-step causal analysis of EHRs to ground decision-making
Source: PLOS Digit Health. 2025 Feb 3;4(2):e0000721. doi: 10.1371/journal.pdig.0000721 (PMC11790099; doi:10.1371/journal.pdig.0000721)
Supplement: S5 Fig — (PDF) [file pdig.0000721.s005.pdf]

Supporting information

S5 Fig Selection flowchart.

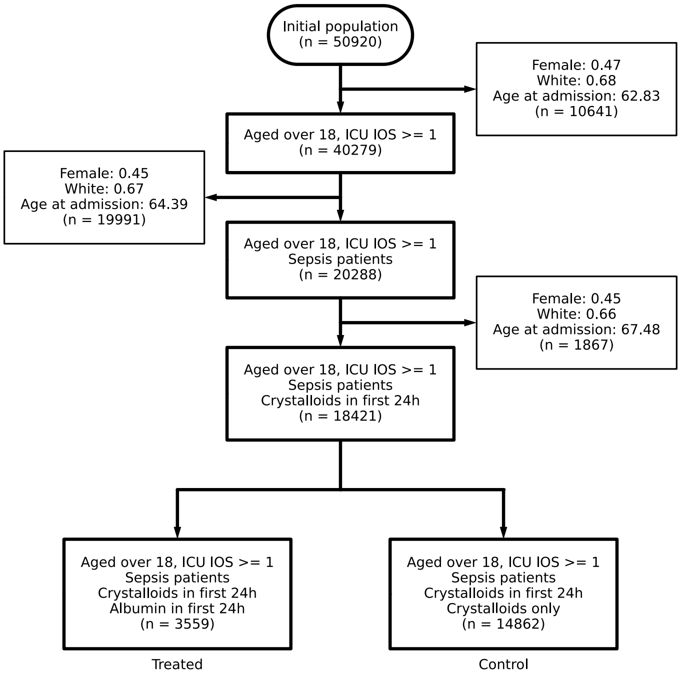

Fig 1. ***Selection flowchart on MIMIC-IV for the emulated trial.***
