# Supplementary material for: Step-by-step causal analysis of EHRs to ground decision-making
Source: PLOS Digit Health. 2025 Feb 3;4(2):e0000721. doi: 10.1371/journal.pdig.0000721 (PMC11790099; doi:10.1371/journal.pdig.0000721)
Supplement: S6 Fig — (PDF) [file pdig.0000721.s006.pdf]

Supporting information

S6 Fig Directed Acyclic Graph.

The expert DAG in figure depicts the known causal links between these variables.

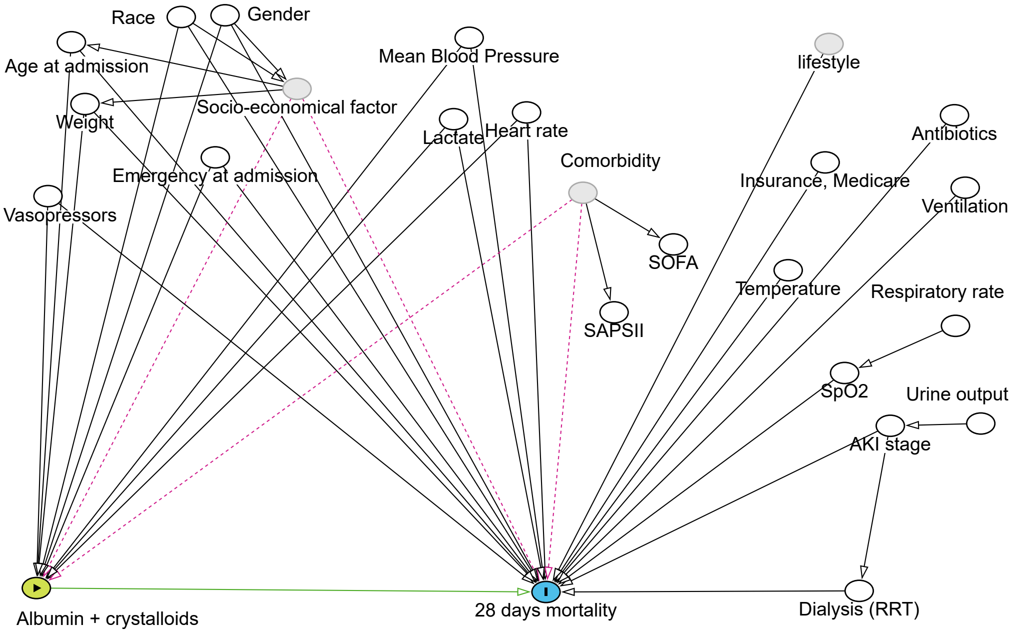

Fig 1. ***Causal graph for the Albumin vs crystalloids emulated trial***

The green arrow indicates the effect studied. Black arrows show causal links known to medical expertise. Dotted red arrows highlight confounders not directly observed. For readability, we draw only the most important edges from an expert point of view. All white nodes correspond to variables included in our study.
