# Supplementary material for: Step-by-step causal analysis of EHRs to ground decision-making
Source: PLOS Digit Health. 2025 Feb 3;4(2):e0000721. doi: 10.1371/journal.pdig.0000721 (PMC11790099; doi:10.1371/journal.pdig.0000721)
Supplement: S7 Fig — (PDF) [file pdig.0000721.s007.pdf]

Supporting information

S7 Fig Complete results for the main analysis.

Compared to Fig 3c), we also report in Fig 1 the estimates for Causal forest estimators and other choices of feature aggregation (first and last).

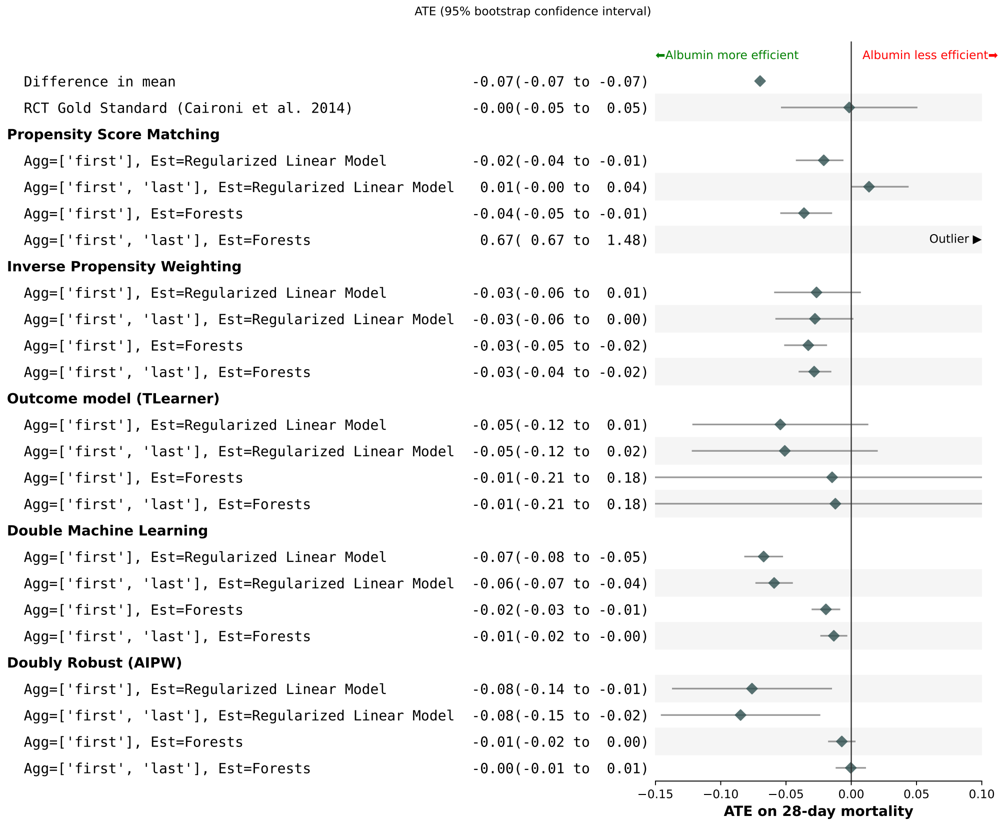

Fig 1. Full sensitivity analysis.

The estimators with forest nuisances point to no effect for almost every causal estimator consistently with the RCT gold standard. Only matching with forest yields an unconvincingly high estimate. Linear nuisance used with doubly robust methods suggest a reduced mortality risk for albumin. The choices of aggregation only marginally modify the results expect for propensity score matching. The green diamonds depict the mean effect and the bar are the 95% confidence intervals obtained by 50 bootstrap repetitions.
