# Supplementary material for: Step-by-step causal analysis of EHRs to ground decision-making
Source: PLOS Digit Health. 2025 Feb 3;4(2):e0000721. doi: 10.1371/journal.pdig.0000721 (PMC11790099; doi:10.1371/journal.pdig.0000721)
Supplement: S8 Fig — (PDF) [file pdig.0000721.s008.pdf]

Supporting information

S8 Fig Complete results for the Immortal time bias.

Compared to Fig 3a), we also report in Fig 1 the estimates for Double Machine Learning, Inverse Propensity Weighting for both Random Forest and Ridge Regression. Feature aggregation was concatenation of first and last for all estimates.

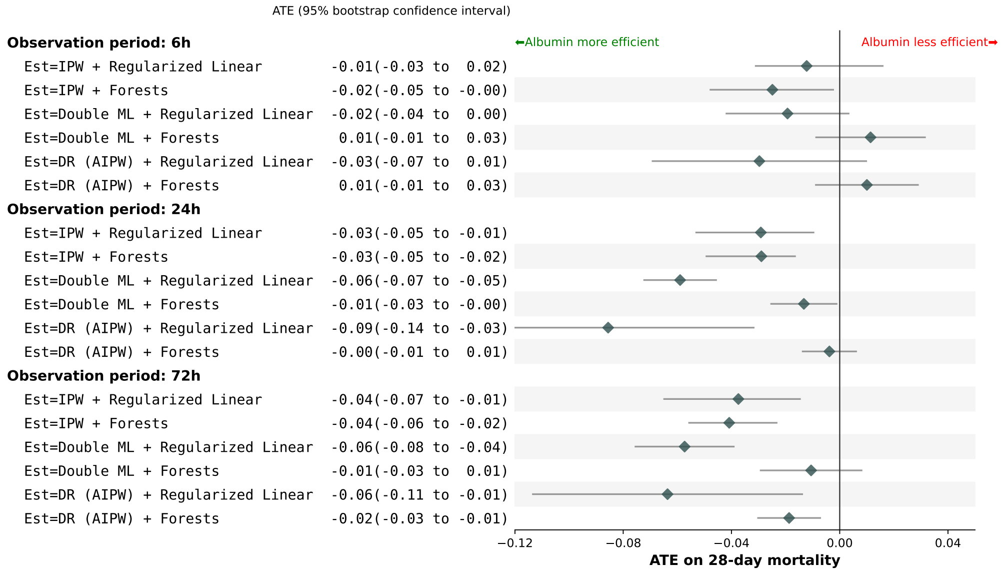

Fig 1. Sensitivity analysis for immortal time bias.
Every choice of estimates show an improvement of the albumin treatment when increasing the observation period, thus increasing the blank period between inclusion and administration of albumin. Aggregation was concatenation of first and last features. The green diamonds depict the mean effect and the bar are the 95% confidence intervals obtained by 50 bootstrap repetitions.
