# Supplementary material for: Step-by-step causal analysis of EHRs to ground decision-making
Source: PLOS Digit Health. 2025 Feb 3;4(2):e0000721. doi: 10.1371/journal.pdig.0000721 (PMC11790099; doi:10.1371/journal.pdig.0000721)
Supplement: S9 Fig — (PDF) [file pdig.0000721.s009.pdf]

Supporting information

S9 Fig Vibration analysis for aggregation.

We conducted a dedicated vibration analysis on the different choices of features aggregation, studying the impact on the estimated ATE. We also studied if some choices of aggregation led to substantially poorer overlap.

We assessed overlap with two different methods. As recommended by [1], we did a graphical assessment by plotting the distribution of the estimated. The treatment model hyper-parameters were chosen by random search, then predicted propensity scores were obtained by refitting this estimator with cross-fitting on the full dataset.

As shown in Fig 1, we did not find substantial differences between methods when plotting graphically the distribution of the estimated propensity score.

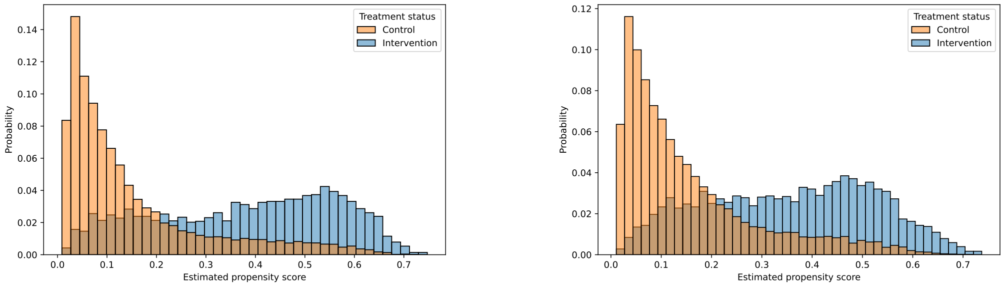

(a) **(b)**

Fig 1. Different choices of aggregation yield qualitatively close distributions of the propensity score.
Fig 1a)a) shows a concatenation of first, last and median measures whereas Fig 1b)b) shows an aggregation by taking the first measure only. The underlying treatment effect estimator is a random forest.

We also used normalized total variation (NTV) as a summary statistic of the estimated propensity score to measure the distance between treated and control population [2]. This statistic varies between 0 – perfect overlap – and 1 – no overlap at all. Fig 2 shows no marked differences in overlap as measured by NTV between aggregation choices, comforting us in our expert-driven choice of the aggregation: a concatenation of first and last feature observed before inclusion time.

References

1. Austin PC, Stuart EA. Moving towards best practice when using inverse probability of treatment weighting (IPTW) using the propensity score to estimate causal treatment effects in observational studies. Statistics in medicine. 2015;34(28):3661–3679.

2. Doutreligne M, Varoquaux G. How to select predictive models for causal inference? arXiv preprint arXiv:230200370. 2023;.

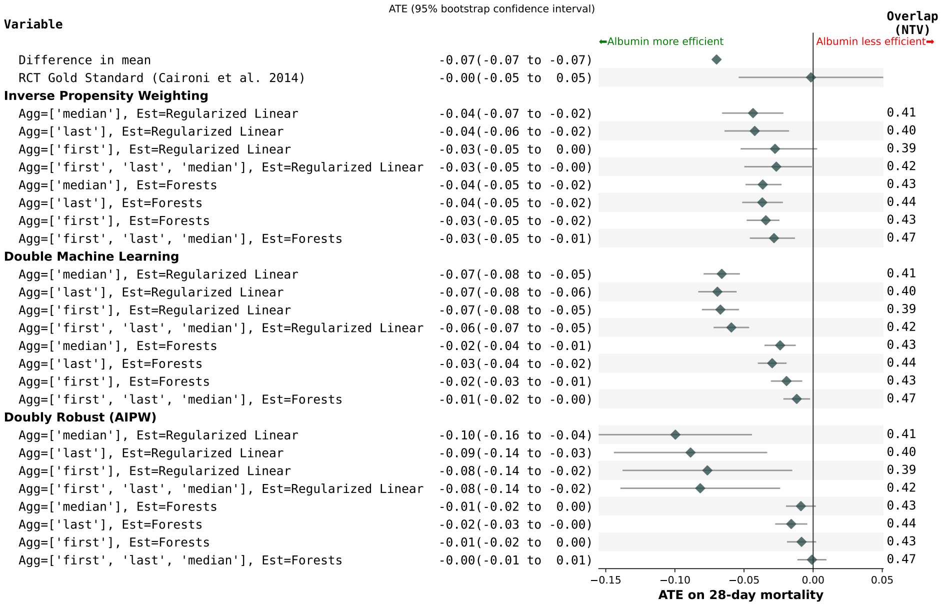

Fig 2. Vibration analysis dedicated to the aggregation choices.
The choices of aggregation only marginally modify the results. When assessed with Normalized Total Variation, the overlap assumption is respected for all our choices of aggregation. The green diamonds depict the mean effect and the bar are the 95% confidence intervals obtained by 50 bootstrap repetitions.
