# Supplementary material for: Step-by-step causal analysis of EHRs to ground decision-making
Source: PLOS Digit Health. 2025 Feb 3;4(2):e0000721. doi: 10.1371/journal.pdig.0000721 (PMC11790099; doi:10.1371/journal.pdig.0000721)
Supplement: S1 Appendix — (PDF) [file pdig.0000721.s010.pdf]

Supporting information

S1 Appendix Assumptions: what is needed for causal inference from observational studies.

The following four assumptions, referred as strong ignorability, are needed to assure identifiability of the causal estimands with observational data with most causal-inference methods [1], in particular these we use:

Assumption 1 (Unconfoundedness)

$$\{Y(0), Y(1)\} \perp\!\!\!\perp A|X \tag{1}$$

This condition –also called ignorability– is equivalent to the conditional independence on the propensity score $e(X) = \mathbb{P}(A = 1|X)$ *[2]:* $\{Y(0), Y(1)\} \perp\!\!\!\perp A|e(X)$.

Unconfoundedness is a strong assumption that might be violated in practice. The existence of residual bias trough unobserved confounders can be mitigated with different strategies. The *omitted variable bias* framework encourages sensitivity analyses allowing to derive bounds on the causal estimate by making assumptions on the strength of association of the omitted variable with both the treatment and the outcome. We refer to [3] for a clear introduction under linear assumption and to [4] for an extension to general non-linear settings. In case of strong unobserved confounders for which proxy variables can be measured, *proximal inference* can be used to obtain identifiability [5]. These methods require expert knowledge to classify the proxy between treatment and outcome proxy, after which a two-stage regression is run to recover the causal effect. Lastly, natural experiments, when available, should be exploited to estimate causal effects without the need of unconfoundedness. Instrumental variable methods exploit randomness influencing the treatment but unrelated to the outcome to simulate a randomized experiment [6, chapter 9]. Regression discontinuity designs leverage discontinuous treatment assignment mechanisms with the assumption of a continuous outcome [6, chapter 5].

Assumption 2 (Overlap, also known as Positivity)

$$\eta < e(x) < 1 - \eta \quad \forall x \in \mathcal{X} \text{ and some } \eta > 0 \tag{2}$$

The treatment is not perfectly predictable. Or in other words, every patient has a chance to be treated and not to be treated. For a given set of covariates, we need examples of both to recover the ATE.

As noted by [7], the choice of covariates X can be viewed as a trade-off between these two central assumptions. A bigger covariate set generally reinforces the ignorability assumption. In the contrary, overlap can be weakened by large $\mathcal{X}$ because of the potential inclusion of instrumental variables: variables only linked to the treatment which could lead to arbitrarily small propensity scores.

Assumption 3 (Consistency) *The observed outcome is the potential outcome of the assigned treatment:*

$$Y = A\,Y(1) + (1 - A)\,Y(0) \tag{3}$$

Here, we assume that the intervention A has been well defined. This assumption focuses on the design of the experiment. It clearly states the link between the observed outcome and the potential outcomes through the intervention [8].

Assumption 4 (Generalization) *The training data on which we build the estimator and the test data on which we make the estimation are drawn from the same distribution, also known as the "no covariate shift" assumption [9].*

References

1. Rubin DB. Causal inference using potential outcomes: Design, modeling, decisions. Journal of the American Statistical Association. 2005;100(469):322–331.

2. Rosenbaum PR, Rubin DB. The central role of the propensity score in observational studies for causal effects. Biometrika;70:41–55.

3. Cinelli C, Hazlett C. Making sense of sensitivity: Extending omitted variable bias. Journal of the Royal Statistical Society Series B: Statistical Methodology. 2020;82(1):39–67.

4. Chernozhukov V, Cinelli C, Newey W, Sharma A, Syrgkanis V. Long story short: Omitted variable bias in causal machine learning. National Bureau of Economic Research; 2022.

5. Tchetgen Tchetgen EJ, Ying A, Cui Y, Shi X, Miao W. An introduction to proximal causal inference. Statistical Science. 2024;39(3):375–390.

6. Wager S. Stats 361: Causal inference; 2020.

7. D'Amour A, Ding P, Feller A, Lei L, Sekhon J. Overlap in observational studies with high-dimensional covariates. Journal of Econometrics. 2021;221(2):644–654.

8. Hernàn MA, Robins JM. Causal inference: What If.; 2020.

9. Jesson A, Mindermann S, Shalit U, Gal Y. Identifying causal-effect inference failure with uncertainty-aware models. Advances in Neural Information Processing Systems. 2020;33:11637–11649.
