# Supplementary material for: Step-by-step causal analysis of EHRs to ground decision-making
Source: PLOS Digit Health. 2025 Feb 3;4(2):e0000721. doi: 10.1371/journal.pdig.0000721 (PMC11790099; doi:10.1371/journal.pdig.0000721)
Supplement: S2 Appendix — (PDF) [file pdig.0000721.s011.pdf]

Supporting information

S2 Appendix Major causal-inference methods: When to use which estimator?

G-formula also called conditional mean regression [1], g-computation [2], or Q-model [3]. This approach is directly modeling the outcome, also referred to as the response surface: $\mu_{(a)}(x) = \mathbb{E}(Y \mid A = a, \mathbf{X} = x)$

Using an outcome estimator to learn a model for the response surface $\hat{\mu}$ (eg. a linear model), the ATE estimator is an average over the n samples:

$$\hat{\tau}_G(f) = \frac{1}{n} \sum_{i=1}^{n} \hat{\mu}(x_i, 1) - \hat{\mu}(x_i, 0) = \frac{1}{n} \sum_{i=1}^{n} \hat{\mu}_{(1)}(x_i) - \hat{\mu}_{(0)}(x_i) \tag{1}$$

This estimator is unbiased if the model of the conditional response surface $\hat{\mu}_{(a)}$ is well-specified. This approach assumes than $Y(a) = \mu_a(X) + \epsilon_a$ with $\mathbb{E}[\epsilon|X] = 0$. The main drawback is the extrapolation of the learned outcome estimator from samples with similar covariates X but different intervention A.

Propensity Score Matching (PSM) To avoid confounding bias, the ignorability assumption (S2Appendix - Assumption 1) requires to contrast treated and control outcomes only between comparable patients with respect to treatment allocation probabilities. A simple way to do this is to group patients into bins, or subgroups, of similar confounders and contrast the two population's outcomes by matching patients inside these bins [4]. However, the number of confounder bins grows exponentially with the number of variables. [5] proved that matching patients on the individual probabilities to receive treatment –propensity scores– is sufficient to verify ignorability. PSM is a conceptually simple method, but has delicate parameters to tune such as choosing a model for the propensity score, deciding what is the maximum distance between two potential matches (the caliper width), the number of matches by sample, and matching with or without replacement. It also prunes data not meeting the caliper width criteria, and suffers from high estimation variance in highly-dimensional data where extreme propensity weights are common. Finally, the simple bootstrap confidence intervals are not theoretically grounded [6] making PSM more difficult to use for applied practitioners.

Inverse Propensity Weighting (IPW)

A simple alternative to propensity score matching is to weight the outcome by the inverse of the propensity score [7]. It relies on a similar idea as matching but automatically builds a balanced population by reweighting the outcomes with the propensity score model $\hat{e}$ to estimate the ATE:

$$\hat{\tau}_{IPW}(\hat{e}) = \frac{1}{n} \sum_{i=1}^{N} \frac{A_i Y_i}{\hat{e}(X_i)} - \frac{(1 - A_i) Y_i}{(1 - \hat{e}(X_i))} \tag{2}$$

This estimate is unbiased if $\hat{e}$ is well-specified. IPW suffers from high variance if some weights are too close to 0 or 1. In high dimensional cases where poor overlap between treated and control is common, one can clip extreme weights to limit estimation instability.

Doubly Robust Learning, DRL also called Augmented Inverse Probability Weighting (AIPW) [8].

The underlying idea of DRL is to combine the G-formula and IPW estimators to protect against a mis-specification of one of them. It first requires to estimate the two nuisance parameters: a model for the intervention $\hat{e}$ and a model for the outcome f. If one of the two nuisance is unbiased, the following ATE estimator is as well:

$$\widehat{\tau}_{AIPW} = \frac{1}{n}\sum_{i=1}^{n}\left(\hat{\mu}_{(1)}\left(x_i\right) - \hat{\mu}_{(0)}\left(x_i\right) + a_i\frac{y_i - \hat{\mu}_{(1)}\left(x_i\right)}{\hat{e}\left(x_i\right)} - \left(1-a_i\right)\frac{y_i - \hat{\mu}_{(0)}\left(x_i\right)}{1-\hat{e}\left(x_i\right)}\right)$$

Moreover, despite the need to estimate two models, this estimator is more efficient in the sense that it converges quicker than single model estimators [9]. For this propriety to hold, one need to fit and apply the two nuisance models in a cross-fitting manner. This means that we split the data into K folds. Then for each fold, we fit the nuisance models on the K-1 complementary folds, and predict on the remaining fold.

To recover Conditional Treatment Effects from the AIPW estimator, [10] suggested to regress the Individual Treatment Effect estimates from AIPW on potential sources of heterogeneity X^{cate}: $\hat{t}au = \arg\min_{\tau\in\Theta}(\hat{\tau}_{AIPW}(X) - \tau(X^{cate}))$ for Θ some class of model (eg. linear model).

Double Machine Learning [11] also known as the R-learner [12]. It is based on the R-decomposition, [13], and the modeling of the conditional mean outcome, $m(x) = \mathbb{E}[Y|X = x]$ and the propensity score, $e(x) = \mathbb{E}[A = 1|X = x]$:

$$y_i - m\left(x_i\right) = \left(a_i - e\left(x_i\right)\right)\tau\left(x_i\right) + \varepsilon_i \quad \text{with } \varepsilon_i = y_i - \varepsilon\left[4_i \mid x_i, a_i\right] \tag{3}$$

Note that we can impose that the conditional treatment effect $\tau(x)$ only relies on a subset of the features, x^{cate} on which we want to study treatment heterogeneity.

From this decomposition, we can derive an estimation of the ATE τ, where the right hand-side term is the empirical R-Loss:

$$\hat{\tau}(\cdot) = \operatorname{argmin}_{\tau}\left\{\frac{1}{n}\sum_{i=1}^{n}\left(\left(y_i - m\left(x_i\right)\right) - \left(a_i - e(x_i)\right)\tau\left(x_i^{cate}\right)\right)^2\right\} \tag{4}$$

The full procedure for R-learning is first to fit the nuisances: $\hat{m}$ and $\hat{e}$. Then, minimize the estimated R-loss eq.4, where the oracle nuisances (e, m) have been replaced by their estimated counterparts $(\hat{e}, \hat{m})$. Minimization can be done by regressing the outcome residuals weighted by the treatment residuals. Finally, get the ATE by averaging conditional treatment effect $\tau(x^{cate})$ over the population.

This estimator has also the doubly robust proprieties described for AIPW. it should have less variance than AIPW since it does not use the propensity score in the denominator.

References

1. Wendling T, Jung K, Callahan A, Schuler A, Shah NH, Gallego B. Comparing methods for estimation of heterogeneous treatment effects using observational data from health care databases. Statistics in medicine. 2018;37(23):3309–3324.

2. Robins JM, Greenland S. The role of model selection in causal inference from nonexperimental data. American Journal of Epidemiology. 1986;123(3):392–402.

3. Snowden JM, Rose S, Mortimer KM. Implementation of G-computation on a simulated data set: demonstration of a causal inference technique. American journal of epidemiology. 2011;173(7):731–738.

4. Stuart EA. Matching methods for causal inference: A review and a look forward. Statistical science: a review journal of the Institute of Mathematical Statistics. 2010;25(1):1.

5. Rosenbaum PR, Rubin DB. The central role of the propensity score in observational studies for causal effects. Biometrika;70:41–55.

6. Abadie A, Imbens GW. On the failure of the bootstrap for matching estimators. Econometrica. 2008;76(6):1537–1557.

7. Austin PC, Stuart EA. Moving towards best practice when using inverse probability of treatment weighting (IPTW) using the propensity score to estimate causal treatment effects in observational studies. Statistics in medicine. 2015;34(28):3661–3679.

8. Robins JM, Rotnitzky A, Zhao LP. Estimation of regression coefficients when some regressors are not always observed. Journal of the American statistical Association. 1994;89(427):846–866.

9. Wager S. Stats 361: Causal inference; 2020.

10. Foster DJ, Syrgkanis V. Orthogonal statistical learning. arXiv preprint arXiv:190109036. 2019;.

11. Chernozhukov V, Chetverikov D, Demirer M, Duflo E, Hansen C, Newey W, et al.. Double/debiased machine learning for treatment and structural parameters; 2018.

12. Nie X, Wager S. Quasi-oracle estimation of heterogeneous treatment effects. Biometrika. 2021;108(2):299–319.

13. Robinson PM. Root-N-consistent semiparametric regression. Econometrica: Journal of the Econometric Society. 1988; p. 931–954.
