# Supplementary material for: Step-by-step causal analysis of EHRs to ground decision-making
Source: PLOS Digit Health. 2025 Feb 3;4(2):e0000721. doi: 10.1371/journal.pdig.0000721 (PMC11790099; doi:10.1371/journal.pdig.0000721)
Supplement: S3 Appendix — (PDF) [file pdig.0000721.s012.pdf]

Supporting information

S3 Appendix Statistical considerations when implementing estimation.

Counterfactual prediction lacks off-the-shelf cross-fitting estimators

Doubly robust methods use cross-fit estimation of the nuisance parameters, which is not available off-the-shelf for IPW and T-Learner estimators. For reproducibility purposes, we did not reimplement internal cross-fitting for treatment or outcome estimators. However, when flexible models such as random forests are used, a fairer comparison between single and double robust methods should use cross-fitting for both. This lack in the scikit-learn API [1] reflects different needs between purely predictive machine learning focused on generalization performances and counterfactual prediction aiming at unbiased inference on the input data.

Good practices for imputation not implemented in EconML

Good practices in machine learning recommend to input distinctly each fold when performing cross-fitting (https://scikit-learn.org/stable/modules/compose.html#combining-estimators). However, EconML estimators test for missing data at instantiation preventing the use of scikit-learn imputation pipelines. We thus have been forced to transform the full dataset before feeding it to causal estimators. An issue mentioning the problem has been filed, so we can hope that future versions of the package will comply with best practices (https://github.com/py-why/EconML/issues/664).

Bootstrap may not yield the most efficient confidence intervals

To ensure a fair comparison between causal estimators, we always used bootstrap estimates for confidence intervals. However, closed form confidence intervals are available for some estimators – see [2] for IPW and AIPW (DRLeaner) variance estimations. These formulas exploit the estimator properties, thus tend to have smaller confidence intervals. On the other hand, they usually do not include the variance of the outcome and treatment estimators, which is naturally dealt with in bootstrapped confidence intervals. Closed form confidence intervals are rarely implemented in any of the packages as Dowhy for the IPW estimator, or in EconML for AIPW.

Bootstrap was particularly costly to run for the EconML doubly robust estimators (AIPW and Double ML), especially when combined with random forest nuisance estimators (from 10 to 47 min depending on the aggregation choice and the estimator). See Table 1 for details.

	estimation method	compute time (seconds)	outcome model	event aggregations
2	LinearDML	1127.98	Forests	['first', 'last']
3	backdoor.propensity_score_matching	199.77	Forests	['first', 'last']
4	backdoor.propensity_score_weighting	86.15	Forests	['first', 'last']
5	TLearner	284.07	Forests	['first', 'last']
6	LinearDRLearner	2855.40	Forests	['first', 'last']
7	LinearDML	49.91	Regularized LR	['first', 'last']
8	backdoor.propensity_score_matching	127.93	Regularized LR	['first', 'last']
9	backdoor.propensity_score_weighting	6.41	Regularized LR	['first', 'last']
10	TLearner	6.84	Regularized LR	['first', 'last']
11	LinearDRLearner	80.75	Regularized LR	['first', 'last']

Table 1. ***Compute times for the different estimation methods with 50 bootstrap replicates.***

References

1. Pedregosa F, Varoquaux G, Gramfort A, Michel V, Thirion B, Grisel O, et al. Scikit-learn: Machine learning in Python. the Journal of machine Learning research. 2011;12:2825–2830.

2. Wager S. Stats 361: Causal inference; 2020.
