# Supplementary material for: Step-by-step causal analysis of EHRs to ground decision-making
Source: PLOS Digit Health. 2025 Feb 3;4(2):e0000721. doi: 10.1371/journal.pdig.0000721 (PMC11790099; doi:10.1371/journal.pdig.0000721)
Supplement: S4 Appendix — (PDF) [file pdig.0000721.s013.pdf]

Supporting information

S4 Appendix Packages for causal estimation in the python ecosystem. We searched for causal inference packages in the python ecosystem. The focus was on the identification methods. Important features were ease of installation, sklearn estimator support, sklearn pipeline support, doubly robust estimators, confidence interva l computation, honest splitting (cross-validation), Targeted Maximum Likelihood Estimation. These criteria are summarized in 1. We finally chose EconML despite lacking `sklearn._BaseImputer` support through the `sklearn.Pipeline` object as well as a TMLE implementation.

The zEpid package is primarily intended for epidemiologists. It is well documented and provides pedagogical tutorials. It does not support sklearn estimators, pipelines and honest splitting.

EconML [1] implements almost all estimators except propensity score methods. Despite focusing on Conditional Average Treatment Effect, it provides all. One downside is the lack of support for scikit-learn pipelines with missing value imputers. This opens the door to information leakage when imputing data before splitting into train/test folds.

Dowhy [2] focuses on graphical models and relies on EconML for most of the causal inference methods (identifications) and estimators. Despite, being interesting for complex inference –such as mediation analysis or instrumental variables–, we considered that it added an unnecessary layer of complexity for our use case where a backdoor criterion is the most standard adjustment methodology.

Causalml implements all methods, but has a lot of package dependencies which makes it hard to install.

Packages	Simple installation	Confidence Intervals	sklearn estimator	sklearn pipeline	Propensity estimators	Doubly Robust estimators	TMLE estimator	Honest splitting (cross validation)
dowhy	✓	✓	✓	✓	✓	✗	✗	✗
EconML	✓	✓	✓	Yes except for imputers	✗	✓	✗	Only for doubly robust estimators
zEpid	✓	✓	✗	✗	✓	✓	✓	Only for TMLE
causalml	✗	✓	✓	✓	✓	✓	✓	Only for doubly robust estimators

Table 1. ***Selection criteria for causal python packages.***

References

1. Battocchi K, Dillon E, Hei M, Lewis G, Oka P, Oprescu M, et al.. EconML: A Python Package for ML-Based Heterogeneous Treatment Effects Estimation; 2019. Available from: https://github.com/py-why/EconML.

2. Sharma A, Kiciman E. DoWhy: An end-to-end library for causal inference. arXiv preprint arXiv:201104216. 2020;.
