# Supplementary material for: Step-by-step causal analysis of EHRs to ground decision-making
Source: PLOS Digit Health. 2025 Feb 3;4(2):e0000721. doi: 10.1371/journal.pdig.0000721 (PMC11790099; doi:10.1371/journal.pdig.0000721)
Supplement: S5 Appendix — (PDF) [file pdig.0000721.s014.pdf]

Supporting information

S5 Appendix Hyper-parameter search for the nuisance models.
We followed a two-step procedure to train the nuisance models (eg. $(\hat{e}, \hat{\mu})$ for the AIPW causal estimator), taking inspiration from the computationally cheap procedure from [1, section 3.3]. First, for each nuisance model, we fit a random parameter search with 5-fold cross validation and 10 iterations on the full dataset. Each iteration fit a model with a random combination of parameters in a predefined grid, then evaluate the performance by cross-validation. The best hyper-parameters $\hat{\lambda}^{\star}$ are selected as the ones reaching the minimal score across all iterations. Then, we feed this parameters to the causal estimator. The single robust estimators (matching, IPW and TLearner) refit the corresponding estimator only once on the full dataset, then estimate the ATE. The doubly-robust estimators use a cross-fitting procedure (K=5) to fit the nuisances then estimate the ATE. Fig 1 illustrates the procedure and Table 1 details the hyper-parameters grid for the random search.

Fig 1. Hyper-parameter search procedure.

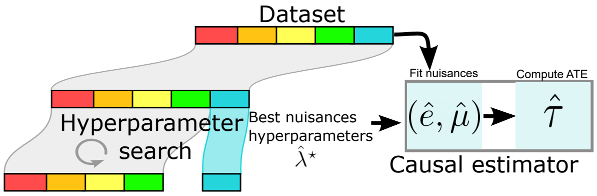

Estimator type	estimator	nuisance	Grid
Linear	LogisticRegression	treatment	{'C': logspace(-3, 2, 10)}
Linear	Ridge	outcome	{'alpha': logspace(-3, 2, 10)}
Forest	RandomForestClassifier	treatment	{'n_estimators': ['10', '100', '200'], 'max_depth': ['3', '10', '50']}
Forest	RandomForestRegressor	outcome	{'n_estimators': ['10', '100', '200'], 'max_depth': ['3', '10', '50']}

Table 1. ***Hyper-parameter grid used during random search optimization.***

References

1. Bouthillier X, Delaunay P, Bronzi M, Trofimov A, Nichyporuk B, Szeto J, et al. Accounting for variance in machine learning benchmarks. Proceedings of Machine Learning and Systems. 2021;3:747–769.
