# Supplementary material for: Step-by-step causal analysis of EHRs to ground decision-making
Source: PLOS Digit Health. 2025 Feb 3;4(2):e0000721. doi: 10.1371/journal.pdig.0000721 (PMC11790099; doi:10.1371/journal.pdig.0000721)
Supplement: S6 Appendix — (PDF) [file pdig.0000721.s015.pdf]

Supporting information

S6 Appendix Deviating from expert ignorability – Impact of smaller confounders sets.

We conducted a dedicated vibration analysis on the different choices of confounders. We created three confounder subsets in addition to all confounders (24 variables): all confounders without antibiotics (Glycopeptides, Beta-lactams, Carbapenems, Aminoglycosides), all confounders without any measurement (weight, lactate, heart rate, spo2, mbp, urine output, temperature, AKI stage, SAPSII, respiratory rate, SOFA), only socio-demographics (admission age, female, emergency admission, insurance–medicare, race).

Fig 3b shows that small deviation from the ignorability assumptions is tolerable: for example, removing antibiotics does not impact the estimate. However, the larger the deviation from the causal graph S6 Fig, the larger the bias compared to the gold-standard. Adjusting only for socio-demographics features is the closest from an unadjusted risk difference, indicating that we lack important confounders on the patient health state. This stability of the treatment effect estimator once sufficient confounders have been included has already been described and suggested as a confounder selection method [1].

References

1. Loh WW, Vansteelandt S. Confounder selection strategies targeting stable treatment effect estimators. Statistics in Medicine. 2021;40(3):607–630.
