# Supplementary material for: Step-by-step causal analysis of EHRs to ground decision-making
Source: PLOS Digit Health. 2025 Feb 3;4(2):e0000721. doi: 10.1371/journal.pdig.0000721 (PMC11790099; doi:10.1371/journal.pdig.0000721)
Supplement: S7 Appendix — (PDF) [file pdig.0000721.s016.pdf]

Supporting information

S7 Appendix Details on treatment heterogeneity analysis.

Detailed estimation procedure

The estimation of heterogeneous effect based on Double Machine Learning adds another step after the computation, regressing the residuals of the outcome nuisance $\tilde{Y} - \mu(X)$ against the residuals of the treatment nuisance $\tilde{A} = A - e(X)$ with the heterogeneity features X_{CATE}. Noting the final CATE model θ, Double ML solves:

$$\arg\min_{\theta} \mathbb{E}_n \left[(\tilde{Y} - \tau(X_C ATE) \cdot \tilde{A})^2 \right]$$

Where $\tilde{Y} = Y - \hat{m}(X)$ and $\tilde{A} = A - \hat{e}(X)$

To avoid the over-fitting of this last regression model, we split the dataset of the main analysis into a train set (size=0.8) where the causal estimator and the final model are learned, and a test set (size=0.2) on which we report the predicted Conditional Average Treatment Effects.

Known heterogeneity of treatment for the emulated trial

[1] observed statistical differences in the post-hoc subgroup analysis between patient with and without septic shock at inclusion. They found increasing treatment effect measured as relative risk for patients with septic shock (RR=0.87; 95% CI, 0.77 to 0.99 vs 1.13;95% CI, 0.92 to 1.39).

[2] conducted a post-hoc subgroup analysis of patients with or without brain injury –defined as Glasgow Coma Scale between 3 to 8–. The initial population was patients with traumatic brain injury (defined as history or evidence on A CT scan of head trauma, and a GCS score <= 13). They found higher mortality rate at 24 months in the albumin group for patients with severe head injuries.

[3] conducted a subgroup analysis on age (<60 vs >60), septic shock and sex. They conclude for increasing treatment effect measured as Restricted Mean Survival Time for Sepsis vs septic shock (3.47 vs. 2.58), for age >=60 (3.75 vs 2.44), for Male (3.4 vs 2.69). None of these differences were statistically significant.

Vibration analysis

The choice of the final model for the CATE estimation should also be informed by statistical and clinical rationals. Fig 1 shows the distribution of the individual effects of a final random forest estimator, yielding CATE estimates that are not consistent with the main ATE analysis. Fig 2 shows that the choice of this final model imposes an inductive bias on the form of the heterogeneity and different sources of noise depending of the nature of the model. A random forest is noisier than a linear model. Fig 2 shows the difference of modelization on the subpopulation of non-white male patients without septic shock. One can see that the decreasing linear trend is reflected by the random forest model only for patients aged between 55 and 80.

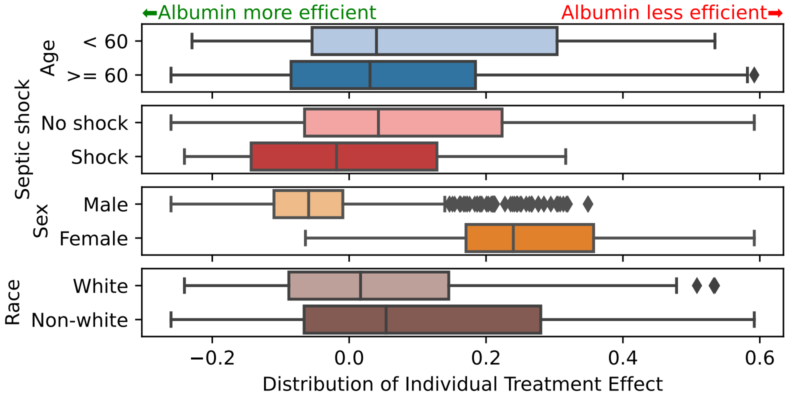

Fig 1. Values of Conditional Average Treatment effects on sex, age, race and pre-treatment septic shock estimated with a final forest estimator.
The CATE are positive for each subgroups, which is not consistent with the null treatment effect obtained in the main analysis. The boxes contain between the 25th and 75th percentiles of the CATE distributions with the median indicated by a vertical line. The whiskers extends to 1.5 the inter-quartile range of the distribution.

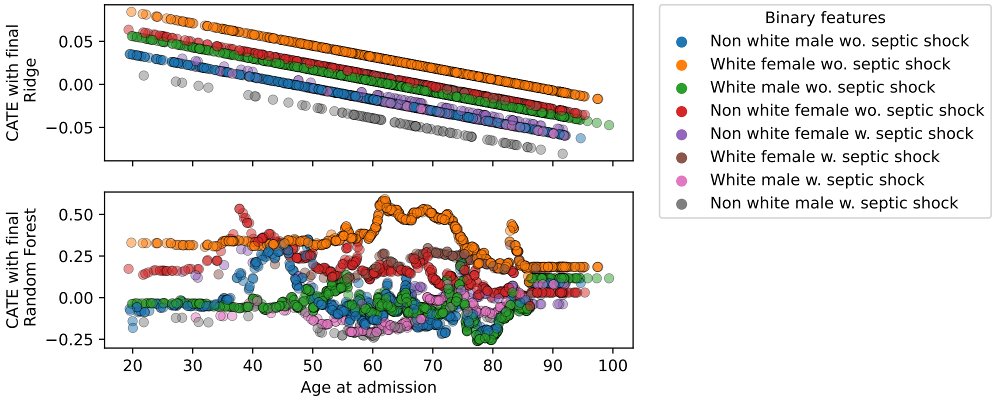

Fig 2. Values of Conditional Average Treatment effects on sex, age, race and pre-treatment septic shock plotted for different ages.
On the top the final estimator is a linear model; on the bottom, it is a random forest. The forest-based CATE displays more noisy trends than the linear-based CATE. This suggest that the flexibility of the random forest might be underfitting the data.

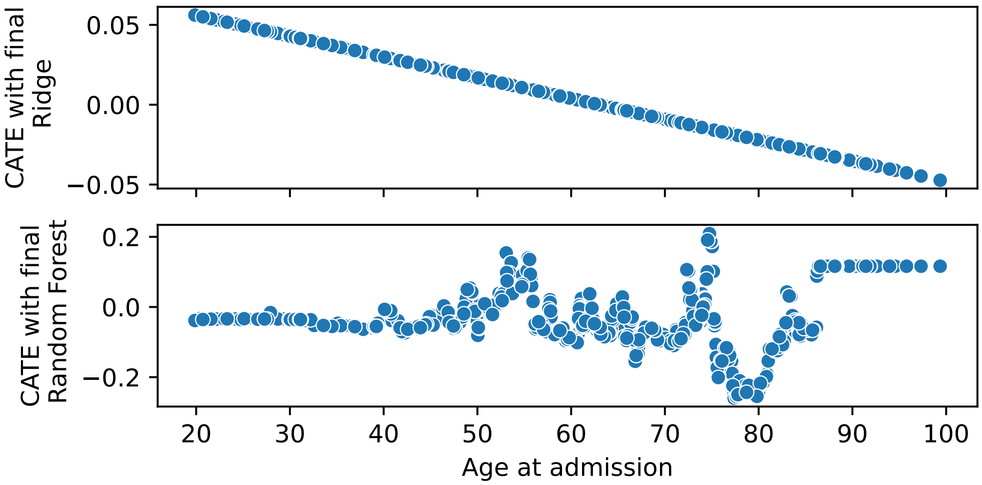

Fig 3. Values of Conditional Average Treatment effects on age, for the subpopulation of white male patients without septic shock.
It is a subset of Fig 2. Contrary to the ridge regression (on top) inducing a nicely interpretable trend, using random forests as the final estimator failed to recover CATE on ages: the predicted estimates do not exhibit any trend and display inconsistently large effect sizes, suggesting data underfitting.

References

1. Caironi P, Tognoni G, Masson S, Fumagalli R, Pesenti A, Romero M, et al. Albumin replacement in patients with severe sepsis or septic shock. New England Journal of Medicine. 2014;370(15):1412–1421.

2. Investigators SS. Saline or albumin for fluid resuscitation in patients with traumatic brain injury. New England Journal of Medicine. 2007;357(9):874–884.

3. Zhou S, Zeng Z, Wei H, Sha T, An S. Early combination of albumin with crystalloids administration might be beneficial for the survival of septic patients: a retrospective analysis from MIMIC-IV database. Annals of intensive care. 2021;11:1–10.
