# Supplementary material for: Step-by-step causal analysis of EHRs to ground decision-making
Source: PLOS Digit Health. 2025 Feb 3;4(2):e0000721. doi: 10.1371/journal.pdig.0000721 (PMC11790099; doi:10.1371/journal.pdig.0000721)
Supplement: S1 Table — (PDF) [file pdig.0000721.s017.pdf]

Supporting information

S1 Table Complete description of the confounders for the main analysis.

	Missing	Overall	Cristalloids only	Cristalloids + Albumin	P-Value
n		18421	14862	3559	
Glycopeptide, n (%)		9492 (51.5)	7650 (51.5)	1842 (51.8)	
Beta-lactams, n (%)		5761 (31.3)	5271 (35.5)	490 (13.8)	
Carbapenems, n (%)		727 (3.9)	636 (4.3)	91 (2.6)	
Aminoglycosides, n (%)		314 (1.7)	290 (2.0)	24 (0.7)	
suspected_infection_blood, n (%)		170 (0.9)	149 (1.0)	21 (0.6)	
RRT, n (%)		229 (1.2)	205 (1.4)	24 (0.7)	
ventilation, n (%)		16376 (88.9)	12931 (87.0)	3445 (96.8)	
vasopressors, n (%)		9058 (49.2)	6204 (41.7)	2854 (80.2)	
Female, n (%)		7653 (41.5)	6322 (42.5)	1331 (37.4)	
White, n (%)		12366 (67.1)	9808 (66.0)	2558 (71.9)	
Emergency admission, n (%)		9605 (52.1)	8512 (57.3)	1093 (30.7)	
Insurance, Medicare, n (%)		9727 (52.8)	7958 (53.5)	1769 (49.7)	
myocardial_infarct, n (%)		3135 (17.0)	2492 (16.8)	643 (18.1)	
malignant_cancer, n (%)		2465 (13.4)	2128 (14.3)	337 (9.5)	
diabetes_with_cc, n (%)		1633 (8.9)	1362 (9.2)	271 (7.6)	
diabetes_without_cc, n (%)		4369 (23.7)	3532 (23.8)	837 (23.5)	
metastatic_solid_tumor, n (%)		1127 (6.1)	1016 (6.8)	111 (3.1)	
severe_liver_disease, n (%)		1289 (7.0)	880 (5.9)	409 (11.5)	
renal_disease, n (%)		3765 (20.4)	3159 (21.3)	606 (17.0)	
aki_stage_0.0, n (%)		7368 (40.0)	6284 (42.3)	1084 (30.5)	
aki_stage_1.0, n (%)		4019 (21.8)	3222 (21.7)	797 (22.4)	
aki_stage_2.0, n (%)		6087 (33.0)	4605 (31.0)	1482 (41.6)	
aki_stage_3.0, n (%)		947 (5.1)	751 (5.1)	196 (5.5)	
SOFA, mean (SD)	0	6.0 (3.5)	5.7 (3.4)	6.9 (3.6)	<0.001
SAPSII, mean (SD)	0	40.3 (14.1)	39.8 (14.1)	42.8 (13.6)	<0.001
Weight, mean (SD)	97	83.3 (23.7)	82.5 (24.2)	86.4 (21.2)	<0.001
temperature, mean (SD)	966	36.9 (0.6)	36.9 (0.6)	36.8 (0.6)	<0.001
mbp, mean (SD)	0	75.6 (10.2)	76.3 (10.7)	72.4 (7.2)	<0.001
resp_rate, mean (SD)	9	19.3 (4.3)	19.6 (4.4)	18.0 (3.8)	<0.001
heart_rate, mean (SD)	0	86.2 (16.3)	86.2 (16.8)	86.5 (14.3)	0.197
spo2, mean (SD)	4	97.4 (2.2)	97.3 (2.3)	98.0 (2.1)	<0.001
lactate, mean (SD)	4616	3.0 (2.5)	2.8 (2.4)	3.7 (2.6)	<0.001
urineoutput, mean (SD)	301	24.0 (52.7)	24.7 (58.2)	21.1 (16.6)	<0.001
admission_age, mean (SD)	0	66.3 (16.2)	66.1 (16.8)	67.3 (13.1)	<0.001
delta mortality to inclusion, mean (SD)	11121	316.9 (640.2)	309.6 (628.8)	365.0 (708.9)	0.022
delta intervention to inclusion, mean (SD)	14862	0.3 (0.2)	nan (nan)	0.3 (0.2)	nan
delta inclusion to intime, mean (SD)	0	0.1 (0.2)	0.1 (0.2)	0.1 (0.1)	0.041
delta ICU intime to hospital admission, mean (SD)	0	1.1 (3.7)	1.0 (3.7)	1.6 (3.4)	<0.001
los_hospital, mean (SD)	0	12.6 (12.5)	12.6 (12.5)	12.9 (12.4)	0.189
los_icu, mean (SD)	0	5.5 (6.7)	5.5 (6.5)	5.5 (7.2)	0.605

Table 1. ***Characteristics of the trial population measured on the first 24 hours of ICU stay.***
Risk scores (AKI, SOFA, SAPSII) and lactates have been summarized as the maximum value during the 24 hour period for each stay. Total cumulative urine output has been computed. Other variables have been aggregated by taking mean during the 24 hour period.
