# Supplementary material for: Step-by-step causal analysis of EHRs to ground decision-making
Source: PLOS Digit Health. 2025 Feb 3;4(2):e0000721. doi: 10.1371/journal.pdig.0000721 (PMC11790099; doi:10.1371/journal.pdig.0000721)
Supplement: Attachment — Submitted filename: Major Recommendations.pdf [file pdig.0000721.s020.pdf]

Recommendations

The manuscript presents a comprehensive framework for robust decision-making based on electronic health record (EHR) data. While the framework is well-structured and addresses important methodological considerations, several aspects need revision to enhance its suitability for publication in reputable journals.

1. Clarity and Organization: The manuscript's structure requires refinement to improve clarity and organization. Each section should flow logically and build upon the previous one. Consider restructuring paragraphs for better coherence and readability.
2. Introduction: The introduction should provide a clear context and motivation for the study, outlining the problem addressed, the research's significance, and its objectives. Including a brief literature review to contextualize the study within existing research would strengthen this section.
3. Methodology: The methodology section needs a more detailed description of data sources, study design, and analytical techniques. Assumptions made during the analysis should be clearly stated and justified to ensure transparency and replicability.
4. Results: Present the results clearly and concisely, using appropriate tables, figures, and descriptive statistics. Provide thorough explanations of the findings and their implications, ensuring they are directly related to the research questions.
5. Discussion: Interpret the results thoroughly and discuss their implications about existing literature. Address any study limitations and suggest future research directions. Emphasize the study's contributions to the field and its potential impact on practice or policy.
6. References: Ensure all references are properly cited and formatted according to the journal's guidelines. Include a comprehensive list of references reflecting relevant literature in the field.
7. Language and Style: Pay close attention to language use, grammar, and writing style, adhering to the journal's formatting and style guidelines. Seek feedback from colleagues or mentors to improve clarity and coherence.
