# Supplementary material for: Step-by-step causal analysis of EHRs to ground decision-making
Source: PLOS Digit Health. 2025 Feb 3;4(2):e0000721. doi: 10.1371/journal.pdig.0000721 (PMC11790099; doi:10.1371/journal.pdig.0000721)
Supplement: Attachment — Submitted filename: Review.docx [file pdig.0000721.s018.docx]

**Comments**

This is an interesting study and the authors have presented a three-step pitfalls retrospective case-control study. However, I believe the paper has some flaws in some text, and I believe this interesting study has not been investigated to its full potential. I've included some comments below.

1) Abstraction: The abstract should be rewritten as a technical summary, while the author's summary should be written in a less technical style for a broader audience.
2) Introduction: The introduction should be improved to better explain the work's motivation and methodology, and the literature review should be more comprehensive, supplementing the advantages and weaknesses of related research. The first sentence of the introduction is hyperbole and not needed.
3) Related works: The literature review should provide a comprehensive overview of the current state of the arts in the field, outlining the strengths and weaknesses of related research.
4) Results: The experimental section needs clarification and more details, and the analysis and explanation of the test results need to be expanded.
