# Supplementary material for: Step-by-step causal analysis of EHRs to ground decision-making
Source: PLOS Digit Health. 2025 Feb 3;4(2):e0000721. doi: 10.1371/journal.pdig.0000721 (PMC11790099; doi:10.1371/journal.pdig.0000721)
Supplement: Attachment — Submitted filename: 2024-02-23_Doutreligne_CI_tutorial_Rebuttal_PLOSdh.docx [file pdig.0000721.s019.docx]

**Journal Requirements:**

1. We ask that a manuscript source file is provided at Revision. Please upload your manuscript file as a .doc, .docx, .rtf or .tex.

We added the source manuscript to the rebuttal.

**Reviewer #1:** This is an interesting study and the authors have presented a three-step pitfalls retrospective case-control study. However, I believe the paper has some flaws in some text, and I believe this interesting study has not been investigated to its full potential. I've included some comments below.

**REPLY: Thank you for your kind words and suggestions.**

1) Abstraction: The abstract should be rewritten as a technical summary, while the author's summary should be written in a less technical style for a broader audience.
**REPLY:** We fully rewrote the abstract and technical summary to match their targeted audience. We refer the reviewer to the revised manuscript for the new versions.

2) Introduction: The introduction should be improved to better explain the work's motivation and methodology, and the literature review should be more comprehensive, supplementing the advantages and weaknesses of related research. The first sentence of the introduction is hyperbole and not needed.
**REPLY:** Thank you for your valuable input. We agree that the motivation for our tutorial can be better displayed. We have extensively reworked the introduction. We removed the first sentence of the introduction and reframed the first paragraph to be much more precise scientifically. We have expended the introduction with new paragraphs on risk scores and the importance of individual treatment model and reformulated the introduction to better present the motivation. Particularly, we displayed the need to study treatment heterogeneity by showcasing the QRISK example that was very briefly presented in the appendix in our initial submission. We detail more extensively previous work and position better this manuscript’s contributions.

We refer the reviewer to the introduction in the revised manuscript: many sentences have been rewritten and sometimes full paragraphs.

3) Related works: The literature review should provide a comprehensive overview of the current state of the arts in the field, outlining the strengths and weaknesses of related research.
**REPLY:** We agree that there is a lot related literature. Thus, we rephrased the introduction to give a more comprehensive overview. We outlined key and recent references from both scientific community that are involved (epidemiology and causal machine learning) for our problem. These modifications can be found in the last part of the introduction, entitled “Multiple Perspectives on Evidence-based Decision Making”.


4) Results: The experimental section needs clarification and more details, and the analysis and explanation of the test results need to be expanded.
**REPLY:** Thank you for your helpful input. We agree that we were very brief on our results. This is to stay within the 4000 word limit of PLOS public health. In the revised manuscript we provide more details in main part of the manuscript: clarification on the patient’s characteristics (right before table 2) and a figure that summarizes the different choices that we tested in the analysis (figure 2), analogous to our original figure 1 showcasing the use of our principles. The supplementary materials detail various parts of the analysis

**Reviewer #2:** The Abstract and Author summary provided by the authors are the same. It would be helpful if the authors can create a distinct non-technical summary under the Author summary section. Other sections are concisely presented.
**REPLY:** Thank you for your review and this suggestion. We rewrote the abstract and technical summary to match their targeted audience. See response to reviewer 1 for the full changes.
