# Supplementary material for: Step-by-step causal analysis of EHRs to ground decision-making
Source: PLOS Digit Health. 2025 Feb 3;4(2):e0000721. doi: 10.1371/journal.pdig.0000721 (PMC11790099; doi:10.1371/journal.pdig.0000721)
Supplement: Attachment — Submitted filename: 2024-06-217_Doutreligne_CI_tutorial_Rebuttal2_PLOSdh.odt [file pdig.0000721.s021.odt]

Reviewer 1 :
Recommendations
The manuscript presents a comprehensive framework for robust decision-making based on
electronic health record (EHR) data. While the framework is well-structured and addresses
important methodological considerations, several aspects need revision to enhance its suitability
for publication in reputable journals.
1. Clarity and Organization: The manuscript's structure requires refinement to improve clarity
and organization. Each section should flow logically and build upon the previous one.
Consider restructuring paragraphs for better coherence and readability.
2. Introduction: The introduction should provide a clear context and motivation for the study,
outlining the problem addressed, the research's significance, and its objectives. Including
a brief literature review to contextualize the study within existing research would
strengthen this section.
3. Methodology: The methodology section needs a more detailed description of data sources,
study design, and analytical techniques. Assumptions made during the analysis should be
clearly stated and justified to ensure transparency and replicability.
4. Results: Present the results clearly and concisely, using appropriate tables, figures, and
descriptive statistics. Provide thorough explanations of the findings and their implications,
ensuring they are directly related to the research questions.
5. Discussion: Interpret the results thoroughly and discuss their implications about existing
literature. Address any study limitations and suggest future research directions. Emphasize
the study's contributions to the field and its potential impact on practice or policy.
6. References: Ensure all references are properly cited and formatted according to the
journal's guidelines. Include a comprehensive list of references reflecting relevant literature
in the field.
7. Language and Style: Pay close attention to language use, grammar, and writing style,
adhering to the journal's formatting and style guidelines. Seek feedback from colleagues or
mentors to improve clarity and coherence.

Authors answer : This second review of our manuscript details seven aspects needing revision. 
However, the seven points risen are quite generic, covering the whole content of the article. It seems like we are given a how-to-guide for scientific writing. We would benefit from more specific commentaries to improve the parts of our manuscript that are unclear.
