# Supplementary material for: Step-by-step causal analysis of EHRs to ground decision-making
Source: PLOS Digit Health. 2025 Feb 3;4(2):e0000721. doi: 10.1371/journal.pdig.0000721 (PMC11790099; doi:10.1371/journal.pdig.0000721)
Supplement: Attachment — Submitted filename: 2024-10-25_Doutreligne_CI_tutorial_third_Rebuttal_PLOSdh.docx [file pdig.0000721.s022.docx]

**Journal Requirements:**

1. Abstract Revision: Please revise the Abstract to more closely reflect the style of other manuscripts published in PLOS Digital Health. Start with clarifying the significance of the problem and why it needs to be studied. Subsequently, explain your methods, summarize the most important results, and conclude with what those mean.
   **REPLY:** We have rephrased the abstract and hope it now matches your requirements.
2. Relevance of QRISK score: The text related to the QRISK score appears tangentially related to the current study. Please explain the connection or consider including it in a distinct manuscript.
   **REPLY:** The QRISK is one concrete example of how a predictive risk can fail. Since it added more confusion than expected, we removed it from the manuscript.
3. Methodology: In Methods, please describe the inclusion and exclusion criteria and how you handled missing data.
   **REPLY:** Thank you for your review. We agree that the methods section needed some clarification. We have thus added a paragraph on missing covariates to the section on confounders: *Missing covariate values might also be a source of confounding. Some statistical estimators (such as forests) can directly incorporate them as supplementary covariates. Others, such as linear models, require imputations. Appendix S3 details general sanity check for imputation strategies when using statistical estimators.*

We also added precisions on how we handle missing data in our application:
*Missing values were median imputed for numerical features, categorical variables were one-hot encoded (thus discarded missing values).*

Concerning inclusion and exclusion criteria, we emphasized the importance of drawing a flowchart :

*A selection flowchart such as in S5 makes inclusion and exclusion choices for PICOT explicit.*

4. Discussion: The Discussion section is focused on methodology; please incorporate a paragraph discussing what your results add to the body of literature about using albumin vs crystalloid and how your results may help the clinicians select the most beneficial treatment.
   **REPLY:** We agree that our discussion section was very brief as we initially planned a tutorial and not a full original publication, but we very well understand that readers might still be interested on our interpretation of the findings. Hence, we expanded the discussion section which now reads as follows:
   *“Since the early 1980ies, researcher investigated the use of colloid fluids in sepsis resuscitation due to their theoretical advantages. However, evidence has long been conflicting. The debate was sparked anew when new synthetic colloid solutions became available, but were later shown to have renal adverse effects (Ref 94). As even large RCTs left unanswered questions, researchers focused on meta-analyses. Here our analysis is in line with the latest two meta-analyses (Ref 94&95), as we found no net benefit for resuscitation with albumin in septic patients overall, but a possible slight benefit for patients with septic shock (see Fig. 4). While regular meta-analyses not utilizing patient-level data are restricted in their sensitivity analyses, our approach offers the benefit to investigate further potential effect modifiers such as age, sex, or race.”*

**Reviewer 6:**

1. The motivation and previous works are well detailed, though the paper is heavily focused on the introduction.
   **REPLY:** We agree that our introduction section is lengthy as we initially planned a tutorial and not a full original publication leading to a paper that is difficult to fit in the common scheme of medical publishing. Given the comments by the editor and yours (see above), we very well understand that readers might still be interested on our interpretation of the findings. Hence, we expanded the discussion section which now reads as follows:

*Since the early 1980ies, researcher investigated the use of colloid fluids in sepsis resuscitation due to their theoretical advantages. However, evidence has long been conflicting. The debate was sparked anew when new synthetic colloid solutions became available, but were later shown to have renal adverse effects (Ref 94). As even large RCTs left unanswered questions, researchers focused on meta-analyses. Here our analysis is in line with the latest two meta-analyses (Ref 94&95), as we found no net benefit for resuscitation with albumin in septic patients overall, but a possible slight benefit for patients with septic shock (see Fig. 4). While regular meta-analyses not utilizing patient-level data are restricted in their sensitivity analyses, our approach offers the benefit to investigate further potential effect modifiers such as age, sex, or race.*

2. A short description of MIMIC is missing.
   **REPLY:** Thank you for your valuable remark. We have added the following paragraph to clarify the content of MIMIC to the readers:

*MIMIC-IV is a publicly available database that contains information from real ICU stays of patients admitted to one tertiary academic medical center, Beth Israel Deaconess Medical Center (BIDMC), in Boston, United States between 2008 and 2019. The data in MIMIC-IV has been previously de-identified, and the institutional review boards of the Massachusetts Institute of Technology (No. 0403000206) and BIDMC (2001-P-001699/14) both approved the use of the database for research. The database contains comprehensive information from ICU stays including vital signs, laboratory measurements, medications, and mortality data up to one year after discharge.*

3. The appendix contains extraneous information, I suggest filtering it out to only relevant information.
   **REPLY:** We agree that the appendix is quite lengthy, which is in part also due to the long review process with several iterations over the manuscript. Thus, we have revised the section and deleted multiple irrelevant parts to make it more succinct.

**Reviewer 7:**

1. One of the goals of the manuscript is to derive clearer assessments of treatment efficacy to limit bias exposure to the best of experts knowledge. However, a critical component of a causal framework is a fine definition of a suitable DAG which to the very least should be compatible with the observed EHR data. A senstitvity analisys with respect to the choice of a DAG should be thus strongly encouraged in any causal framework and deserves a proper focus in a manuscript such as the one proposed by the authors.
   **REPLY:** Thank you for your concern about a valid DAG. We fully agree that a valid expert-derived DAG is crucial for proper causal inference. This is why we already performed a vibration analysis excluding certain variables from the expert DAG (this is the paragraph “Confounder choice” in the “vibration analysis” section). We added a sentence underlying that the observed bias in this analysis is introduced by the incompleteness of the DAG :
   *This is consistent with the literature enhancing the importance of a clinically valid DAG (Ref 48).*
   A more subtle exploration of the effect of DAG variations on bias would be an interesting study but we think it should be left for future work.
2. Unconfoundedness is a strong a hypothesis that rarely can be enforced in real scenarios. There is however an increasing body of litetarure attempting to solve partial identifiability and cases were causal sufficiency can not be granted. I suggest the authors to further expand their mansucripts to included these concepts.
   **REPLY:** Thank you for this remark. Interesting works indeed exist to mitigate real world cases where unconfoundedness might not apply. We expanded the identification paragraph in the framework section to add some useful references in case where unobserved counfounders are known to the analyst :

*Unconfoundedness --inclusion of all confounders in the analysis-- is a strong hypothesis that can be hard to obtain in real applications. In these cases, sensitivity analyses for omitted variable bias allow to test the robustness of the results to missing confounders (Ref 67), proximal inference can be used to leverage proxy of unobserved confounders (Ref 68), and the presence of natural experiment might identify the desired causal effect without unconfoundedness (Ref 69, Chapter 5, 9).*

3. There is seems to be some confusion between causal ad statistical concepts, specifically in line 180. The literature consensus is that causal estimands can be evaluated via statistical estimators through identification.
   **REPLY:** We thank the reviewer for this remark, we split the causal and statistical estimation in two different sections for clarity and moved the causal estimator paragraph in the previous section on identification.
4. There has been an issue in the latex export of the paper, please remove the "Missing charachter" warning between the bibliography and the appendix
   **REPLY:** Thank you for your keen eye by spotting this error which we have now fixed.
5. I suggest the authors to split the causal inference section from the statistical estimations section for clarity
   **REPLY:** We thank the reviewer for this remark. We split the causal and statistical estimation in two different sections for clarity.
